# Genomics-based identification of a cold adapted clade in *Deinococcus*

**DOI:** 10.1186/s12915-024-01944-8

**Published:** 2024-07-02

**Authors:** Liang Shen, Jiayu Hu, Luyao Zhang, Zirui Wu, Liangzhong Chen, Namita Paudel Adhikari, Mukan Ji, Shaoxing Chen, Fang Peng, Yongqin Liu

**Affiliations:** 1 College of Life Sciences, Anhui Normal University, Wuhu, 241000 China; 2 Anhui Provincial Key Laboratory of Molecular Enzymology and Mechanism of Major Diseases, and Auhui Provincial Engineering Research Centre for Molecular Detection and Diagnostics, Anhui Normal University, Wuhu, 241000 China; 3 State Key Laboratory of Tibetan Plateau Earth System, Environment and Resources (TPESER), Institute of Tibetan Plateau Research, Chinese Academy of Sciences, Beijing, 100101 China; 4 Center for the Pan-Third Pole Environment, Lanzhou University, Lanzhou, 730000 China; 5 China Center for Type Culture Collection (CCTCC), College of Life Sciences, Wuhan University, Wuhan, 430072 People’s Republic of China

**Keywords:** Ecological unit, Genomics, Cold habitat, Environmental adaptation, *Deinococcus*

## Abstract

**Background:**

Microbes in the cold polar and alpine environments play a critical role in feedbacks that amplify the effects of climate change. Defining the cold adapted ecotype is one of the prerequisites for understanding the response of polar and alpine microbes to climate change.

**Results:**

Here, we analysed 85 high-quality, de-duplicated genomes of *Deinococcus*, which can survive in a variety of harsh environments. By leveraging genomic and phenotypic traits with reverse ecology, we defined a cold adapted clade from eight *Deinococcus* strains isolated from Arctic, Antarctic and high alpine environments. Genome-wide optimization in amino acid composition and regulation and signalling enable the cold adapted clade to produce CO_2_ from organic matter and boost the bioavailability of mineral nitrogen.

**Conclusions:**

Based primarily on in silico genomic analysis, we defined a potential cold adapted clade in *Deinococcus* and provided an updated view of the genomic traits and metabolic potential of *Deinococcus*. Our study would facilitate the understanding of microbial processes in the cold polar and alpine environments.

**Supplementary Information:**

The online version contains supplementary material available at 10.1186/s12915-024-01944-8.

## Background

Understanding the genetic make-up of microbes in a given environmental condition is one of the fundamental tasks of environmental microbiology and climate change microbiology in particular [1–3]. Polar and alpine environments are more sensitive to climate change, and microbes in these environments feedback and amplify the effects of climate change, for example, by producing and consuming greenhouse gases like CO_2_, CH_4_ and N_2_O [4, 5]. As important as polar and alpine microbes are, there is no unifying concept of polar and alpine microbiology yet. One challenge is to sort the diversity of polar and alpine microbes into ecologically meaningful units and to define the cold-adapted ecotypes.

The cold polar and alpine environments are colonised by or preserve a high diversity of microbes [6, 7]. These can be recovered by traditional pure-cultivation or obtained by direct sequencing from environmental samples, providing isolates and metagenomes [8, 9]. Individual genomes, MAGs and metagenomes have been used to understand the phenotypic and genetic characteristics of microbial adaptation to polar and alpine environments [10, 11].

Unlike temperate or thermal environments, the low temperature condition of polar and alpine environments selects psychrophiles; on the other hand, low temperature conditions are also ideal for the preservation of DNA and cells [12]. For example, most rDNA clones from the snow around Russian Antarctic stations were not alive and/or active [13]. And some microbes could also survive through phenotypic plasticity without genetic change; for example, snow-bacteria with different genetic backgrounds from glaciers on the Tibetan Plateau shifted their growth temperature downward to adapt to cold [14]. Although mesophiles could be phenotypically conserved to psychrophiles in a short time, phenotypic plasticity in general does not always facilitate genetic adaptation [15].

The bacterial genus *Deinococcus* (phylum Deinococcus-Thermus; class Deinococcus; order Deinococcales; family Deinococcaceae) is a coccoid or rod-shaped, non-sporulating bacterium characterised by a high tolerance to ionising radiation, UV radiation, desiccation, oxidants, low temperature and many other harsh environmental factors [16, 17]. *Deinococcus* have been isolated from a wide range of habitats such as processed meat, soil, desert, vegetation, freshwater, faeces and thermal springs, spanning from the mid-latitude regions to polar and high alpine reaches [18, 19]. Strains of *Deinococcus* derived from different sources are mixed in the phylogenetic tree, making it difficult to define its natural habitat ecotype. One case is for identification of the cold adapted *Deinococcus* ecotype, as the eight *Deinococcus* strains isolated from Arctic, Antarctic and high alpine environments located in three different positions in the phylogenetic tree. We speculate that with the well-established genomic proxy of cold adaptation and multiple genome sequences and the associated phenotypic data, it is now possible to define the polar and alpine-environment ecotype of *Deinococcus* [18–20].

In this study, we sequenced two genomes and utilised the existence of 83 *Deinococcus* genomes to identify the polar and alpine ecotype from *Deinococcus.* Combining genomic traits, sources of isolation and physiological information, we identified a clade that includes four Antarctic isolates and showed consistency in genomic and phenotypic traits based on reverse ecology theory.

## Results

### Phylogeny and general genomic features of Deinococcus

**Fig. 1:**
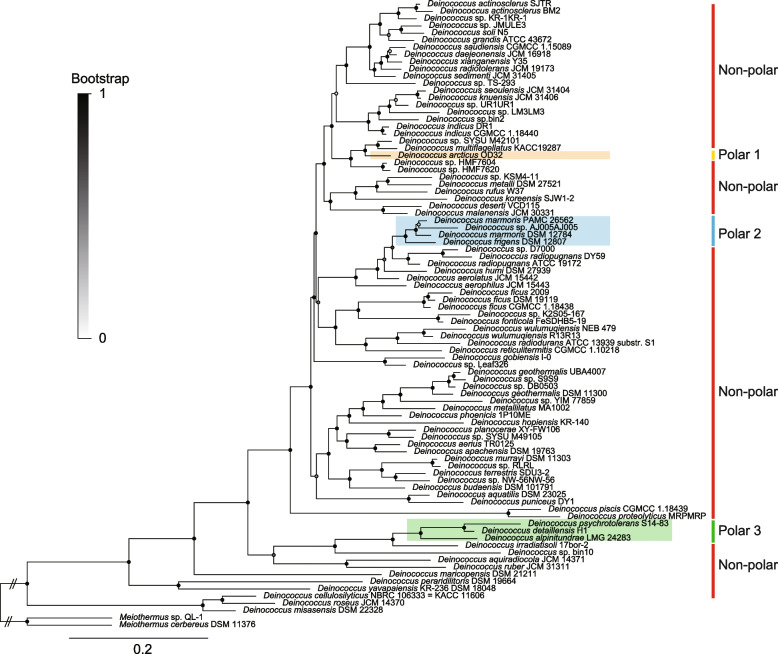
*Deinococcus* phylogeny and distribution of Arctic and Antarctic isolates in the tree rooted by *Meiothermus cerbereus* DSM 11376 and *Meiothermus* sp. QL-1. The tree was sorted with increasing node order; the Arctic isolate (*D. arcticus* OD32) is in the upper part of the tree; four Antarctic isolates are in the middle (*D. marmoris* PAMC 26562, *D. marmoris* DSM 12784, *Deinococcus* sp. AJ005AJ005, *D. frigens* DSM 12807); two Antarctic isolates and one alpine isolate at the bottom (*D. psychrotolerans* S14-83, *D. detaillensis* H1, *D. alpinitundrae* LMG 24283) of the tree. These three clades are referred to as the polar 1 (*n* = 1), polar 2 (*n* = 4) and polar 3 (*n* = 3) for brevity in the following sections. The four isolates from Polar 2 and the two from Polar 3 formed monophyletic clades. All the other genomes were set as non-polar group. The maximum likelihood phylogenomic tree was constructed using PhyloPhlAn 3.0. Dots at nodes indicate bootstrap. Bar 0.2 indicates accumulated changes per amino acid

The phylogeny of *Deinococcus* showed that most of the isolates from different habitats formed mixed clusters with each other (Fig. 1, Additional file 1: Table S1). For example, isolates from polar and high alpine environments (i.e. the Arctic, Antarctic and Mount Evans) were distributed across the root, middle and top of the sorted phylogenomic tree (Fig. 1). The Arctic isolate, *D. arcticus* OD32 (referred to as polar 1 in Fig. 1), was in the middle of a clade containing strains from freshwater, evaporation cores and vegetation (Fig. 1 and Additional file 1: Table S1). The six Antarctic isolates with one high alpine derived strain in the root and middle parts of the tree, termed polar groups 2 and 3 in Fig. 1. The four isolates from polar 2 (*D. marmoris* PAMC 26562, *D. marmoris* DSM 12784, *Deinococcus* sp. AJ005AJ005, *D. frigens* DSM 12807) and the three from polar 3 (*D. psychrotolerans* S14-83, *D. detaillensis* H1 and *D. alpinitundrae* LMG 24283) were clustered together forming monophyletic clades (Fig. 1). Not only the polar and alpine strains were distantly distributed in the phylogenomic tree, the MAGs (*Deinococcus* sp. bin2 and *Deinococcus* sp. bin10) from the same sample were also distributed separately in the tree and had a large phylogenetic distance (Fig. 1). This situation paralleled the finding that *Exiguobacterium* MAGs from the New York subway system belonged to completely different ecotypes [21].

### General genomic features and *pan*-genome of Deinococcus

**Fig. 2:**
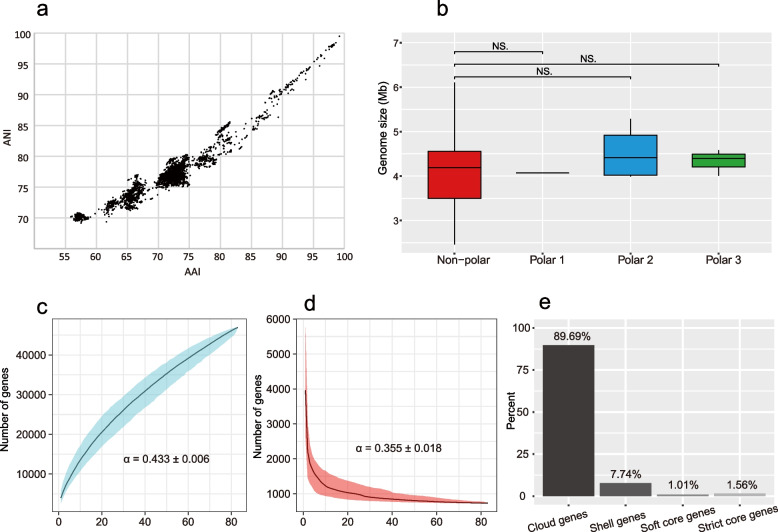
Genetic relatedness and pangenome of *Deinococcus*. **a** Distribution and correlation of ANI and AAI. **b** Comparison of genome size. **c** Rarefaction curve for the accumulation of pan genes. **d** Rarefaction curve for the reduction of core genes. **e** Summary statistics of the 46,923 pan genes of *Deinococcus* – cloud genes: 0% ≦ strains < 15%; strict core genes: strains = 100%; shell genes: 15% ≦ strains < 95%; soft core genes: 95% ≦ strains < 99%. The curves were fitted to median values of 1000 permutations. The dark lines in panels a and b indicate median values and the shading indicates the 95% confidence interval. *α* = 0.443 ± 0.006 in **a** and *α* = 0.355 ± 0.018 in **b**, indicating an open pan- and core genome of *Deinococcus*

Whole-genome similarity metric showed that the ANI (average nucleotide identity) and AAI (average amino acid identity) between the 85 genomes were 69.18 ~ 99.49 and 55.87 ~ 99.18, respectively (Fig. 2a). The genome sizes of *Deinococcus* ranged from 2.77 Mb (*D. murrayi* DSM 11303, isolated from a hot spring) to 6.65 Mb (*D. hopiensis* KR-140, isolated from desert soil), with a mean value of 4.15 Mb. Genome sizes did not differ significantly between the polar and non-polar groups (Fig. 2b). Although the metagenome assembled genome (*Deinococcus* sp. bin10) had the smallest calculated genome size of 2.46 Mb, it was not used to indicate the minimum genome size of *Deinococcus*, because genome completeness was estimated based on housekeeping marker genes by CheckM, which may not fully reflect the size of the non-pure culture derived genome [22].

**Fig. 3:**
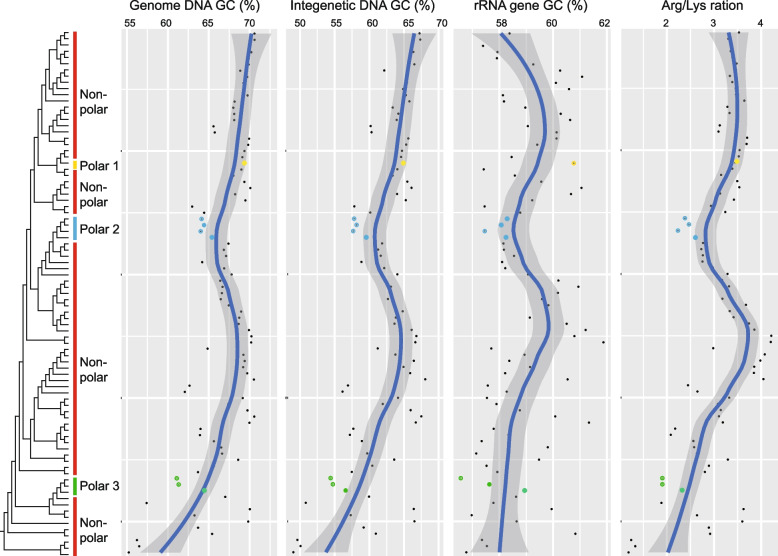
Cladogram and dot plots showing the trends of genome-wide GC content, intergenic GC content, rRNA GC content and Arg/Lys ration in *Deinococcus*. A clear drop in the Arg/Lys ration with respect to close neighbours (the six genomes, *Deinococcus* sp. D7000, *D. radiopugnans* DY59, *D. radiopugnans* ATCC 19172, *D. humi* DSM 27939, *D. aerolatus* JCM 15442 and *D. aerophilus* JCM 15443, in the same clade of polar 2 as shown in Fig. 1) of polar group 2 was indicated using an arrow

The 85 *Deinococcus* genomes constituted an ‘open’ pan-genome, as indicated by the alpha value of 0.443 ± 0.006, which, being < 1, is indicative of this feature (Fig. 2c) [23]. It was predicted that approximately 325 genes will be found once a new genome has been added to the pan-genome. Meanwhile, around 3 core genes will be excluded following the addition of a new genome (Fig. 2d). Of the 46,923 genes in the pan-genome, most (89.69%) were shared by < 15% of isolates, forming the cloud genes (Fig. 2e). The shell genes (present in 15% ≦ isolates < 95%) made up 7.74% of the pan-genome, and the resting 2.57% was composed of the core genes (both strict and soft genes, present in ≥ 95% strains, Fig. 3c).

The genome-wide GC (guanine + cytosine) contents of *Deinococcus* varied dramatically, with the lowest being 55.22% (*D. misasensis* DSM 22328, isolated from freshwater), the highest being 70.60% (*D. actinosclerus* BM2, isolated from soil) and the average being 66.94% (Fig. 3, Table S1). Genomes with the lowest GC content (including genomic DNA, intergenic DNA and rRNA DNA) were in the lower part of the phylogenomic cladogram, highlighting a trend that the variation in genome GC content converged from the root to the top of the tree, sorted by increasing node order (Fig. 3). Such a trend was not seen in the distribution of complete metabolic pathways along the phylogenomic cladogram (Additional file 2: Fig. S1). The predicted complete metabolic pathways of *Deinococcus* ranged from 206 to 300, and isolates that conducted more metabolic reactions were concentrated in the upper part of the tree (Fig. S1). In the upper part of the tree, the two main peaks in the number of metabolic pathways each contained four and six isolates derived from a diverse range of environments such as soil, sediment, vegetation and seawater (Additional file 2: Fig. S1). Apart from the fluctuation in GC content, there were troughs in GC content in polar group 2, and there is a clear decrease in Arg/Lys ratio in this group (Fig. 3).

### Signature of cold adaptation of Deinococcus isolates from polar environments

**Fig. 4:**
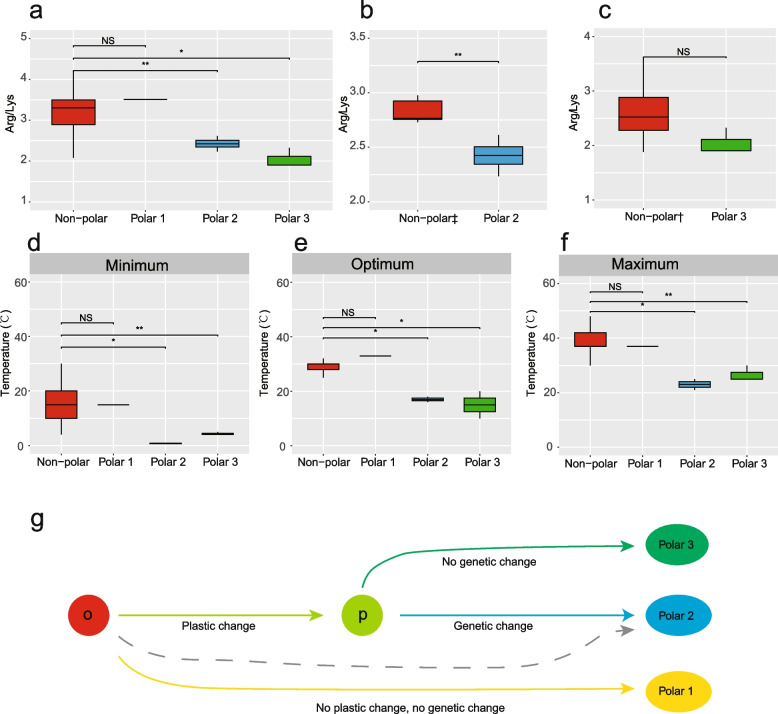
Comparison of Arg/Lys ratio and growth temperature profile between *Deinococcus* groups. **a** Comparison of Arg/Lys ratio between the polar groups and the reference group. **b** Comparison of Arg/Lys ratio between polar group 2 and six non-polar genomes in the same clade. **c** Comparison of Arg/Lys ratio between polar group 3 and four non-polar genomes in the same clade; comparison of **d** Minimum. **e** Optimum and **f** Maximum growth temperature profile between the polar groups and the non-polar group. ^‡^This non-polar group including six genomes, *Deinococcus* sp. D7000, *D. radiopugnans* DY59, *D. radiopugnans* ATCC 19172, *D. humi* DSM 27939, *D. aerolatus* JCM 15442 and *D. aerophilus* JCM 15443; ^†^this non-polar group including four genomes, *D. irradiatisoli* 17bor-2, *Deinococcus* sp. bin10, and *D. aquiradiocola* JCM 14371; *p < 0.05, **p < 0.01, NS, not significant. **g** Three groups of *Deinococcus* strains isolated from cold environments: polar group 1 had not processed plastic (p) or genetic change from ancestors (o); polar group 2 had plastic and genetic change or evolved directly from ancestor (grey dotted line); polar group 3 had processed plastic change but not genetic change

Compared with the non-polar group, a significant decrease in the genome-wide Arg/Lys ratio was identified in polar group 2 and 3 (Fig. 4a, Wilcoxon test, *p* < 0.05), but not in polar group 1. When compared with their closely related genomes in the same clade, the trend of increasing Arg/Lys ratio was still observed for polar group 2 (Fig. 4b, Wilcoxon test, *p* < 0.05), but not in polar group 3 (Fig. 4c, Wilcoxon test, *p* > 0.05). The decrease in genomic GC content was significant in polar group 2 compared to all non-polar genomes and their closely related genomes in the same clade. For the late case, the significance was cancelled out in polar group 3, similar to the situation for the Arg/Lys ratio (Additional file 2: Fig. S2). Except for polar group 1, both polar groups 2 and 3 had lower minimum, optimum and maximum growth temperatures than the reference group (Fig. 4d–f, Wilcoxon test, *p* < 0.05). Then, three groups of *Deinococcus* strains isolated from cold environments were featured out: polar group 1 had not processed plastic or genetic changes from ancestors; polar group 2 had processed plastic and genetic changes, or direct from *Deinococcus* ancestor via genetic selection; polar group 3 had processed plastic changes but not genetic changes for cold adaptation (Fig. 4g).

### Biogeochemical cycle processes of Deinococcus

**Fig. 5:**
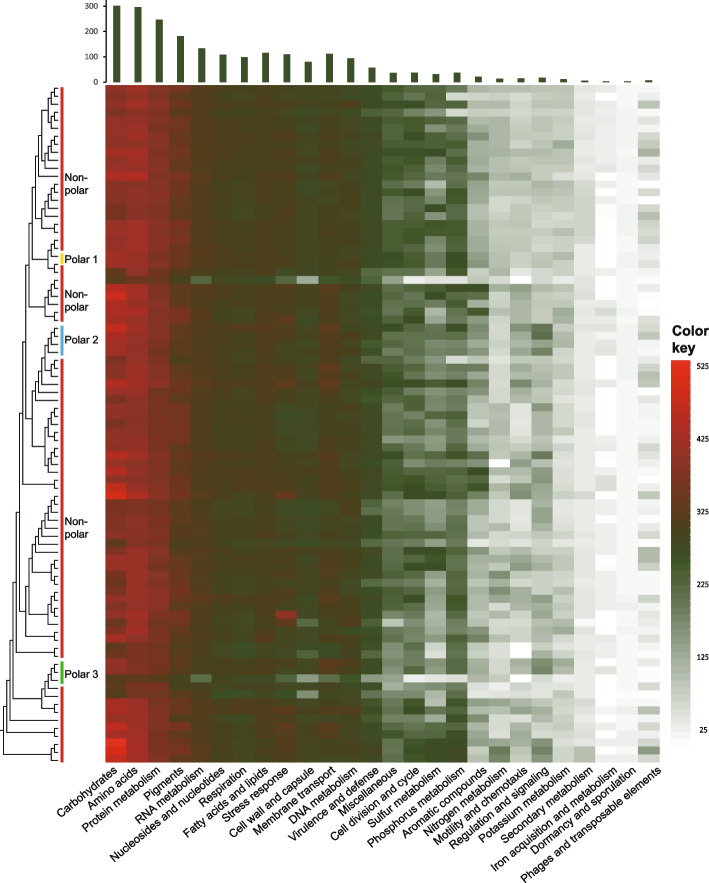
Cladogram and heatmap showing the distribution of functional categories of *Deinococcus*. The left panel is the cladogram with grouping profile of *Deinococcus*, the upper panel is bar chart showing average genes in each category of *Deinococcus* and the middle panel is heatmap showing the distribution of functional categories of each *Deinococcus* genome

The polar isolates had a similar overall distribution of functional categories, with most of their genomes devoted to ‘carbohydrates metabolism’, ‘amino acids and derivatives metabolism’ and ‘protein metabolism’ (Fig. 5). The function category ‘regulation and cell signalling’ was significantly enriched in polar group 2 compared to other *Deinococcus*, with an average of 31 vs. 16 (Fig. 5).

**Fig. 6:**
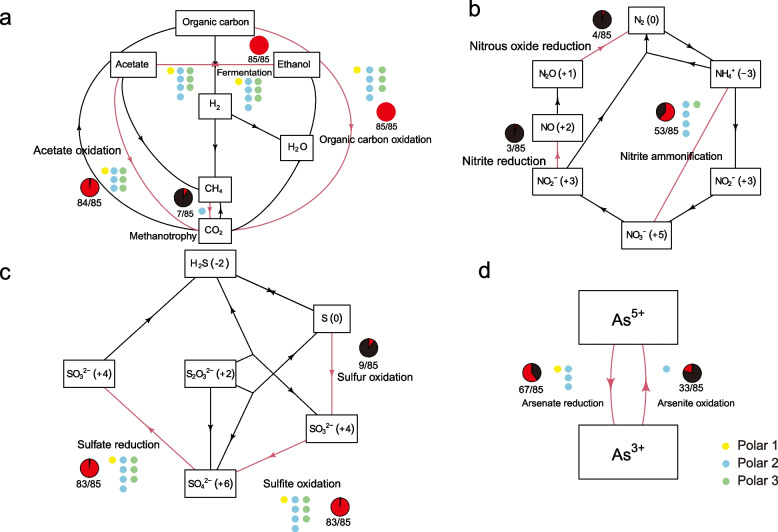
Summary scheme of carbon, nitrogen, sulphur and arsenate cycling processes of *Deinococcus*. Each arrow represents a single transformation to step within a cycle; arrows in red indicate at least one isolate was positive for the reactions, and red parts of the pie charts indicate the percentage of isolates that can conduct these reactions. **a** Carbon cycle scheme showing that *Deinococcus* are typical heterotrophic bacteria characterised by being capable of utilizing organic carbon, fermenting ethanol and oxidising acetate, but not fixing CO_2_. Seven of the 85 isolates were predicted to be able to utilise CH_4_. **b** Nitrogen cycle scheme. **c** Sulphur cycle scheme. The chemical states of the nitrogen or sulphur atoms in **b** and **c** are indicated by the numbers in parentheses on the right of the nitrogen or sulphur-containing compounds. **d** Arsenate oxidation–reduction scheme showing that *Deinococcus* can drive the transformation between As^5+^ and As^3+^. Yellow, blue and green dots represent genomes from polar 1, 2 and 3 that were positive in the relevant reactions

Recently developed standardised tools for analysing genomic data allowed us to read the genomic blueprints of *Deinococcus* and reconstruct their roles in biogeochemical processes [24]. The carbon cycle scheme indicated that *Deinococcus* are typical heterotrophic microorganisms that use organic carbon, including acetate and ethanol, as their main energy source (Fig. 6a). Seven isolates, including one from polar group 2, were predicted to be able to oxidise CH_4_, four of which (*D. apachensis* DSM 19763, *D. aerius* TR0125, *D. planocerae* XY-FW106 and *Deinococcus* sp. SYSU M49105) were clustered together in the phylogenomic tree (Fig. 1 and Fig. 6a). Most of the reactions in the nitrogen cycle were absent in *Deinococcus* (shared less than 5% of isolates), but over 60% (53/85) of the isolates were predicted to be able to conduct nitrite ammonification, all the polar 2 isolates were positive in this reaction (Fig. 6b). In the sulphur cycle, *Deinococcus* were predicted to be able to oxidise SO_3_^2−^ to SO_4_^2−^ and redox SO_4_^2−^ back to SO_3_^2−^, and nine isolates were likely to be able to conduct the oxidisation of S^0^ to SO_3_^2−^ (Fig. 6c). Of the other cycles besides carbon, nitrogen and sulphur, we found that *Deinococcus* can drive the transformation between As^5+^ and As^3+^, with ~ 80% of isolates conducting the reduction and ~ 40% the oxidisation (Fig. 6d).

## Discussion

### Genomic features of Deinococcus and defining the cold adapted clade

In this study, we present an updated view of the genomic features of the genus *Deinococcus*. The first reported genome of *Deinococcus* was that of *D. radiodurans* R1, which is about 3.28 Mb in size and has a 66.94% GC content [25]. From the current dataset containing 85 nonredundant high-quality genomes contributed by the community worldwide, it was found that *Deinococcus* has an open pan-genome and a strict core genome with 733 genes. The genome size ranging from 2.77 Mb to 6.65 Mb and GC content ranging from 55.22 to 70.60%. Generally, in harsher environments (e.g. dry, cold and hot), the genome is expected to be smaller in size and more compact with functional genes [26, 27]. This trend was identified with strain *D. murrayi* DSM 11303 isolated from a hot spring, which had the smallest genome size of 2.77 Mb, but not in the polar and alpine genomes. Trends in genome size of cold adapted bacteria are not yet clear, and there is currently no general trend in genome GC content and indicator genes of cold adaptation [28, 29].

However, the optimisation of genome-wide amino acid composition to achieve low temperature activity (e.g. reduction of Arg and increase of Lys) seems to apply to all well-defined cold adapted bacteria and archaea across broad taxa [11, 21, 30]. This is reasonable because bacterial cells are at complete thermal equilibrium with their environment; whatever the other environmental conditions (nutritional status, salinity and pH, etc.) in which psychrophiles live, all the proteins must be adapted to the cold to enable an overall level of activity that is sufficient for growth at low temperature [29]. According to the principle of reverse ecology, the bacterial genome contains identifiable adaptation traits to its native environment. Using the Arg/Lys ratio as a proxy for cold adaptation, four genomes (*D. marmoris* PAMC 26562, *D. marmoris* DSM 12784, *Deinococcus* sp. AJ005AJ005, *D. frigens* DSM 12807) in polar 2 showed a clear signature of cold adaptation. The Arctic isolate *D. arcticus* OD32 showed no significant change in the Arg/Lys ratio. Although the polar group 3 (*D. psychrotolerans* S14-83, *D. detaillensis* H1, *D. alpinitundrae* LMG 24283) had a lower Arg/Lys ratio in comparison to all the non-polar genomes, this trend might only be caused by phylogenetic divergence [31], as the trend was not significant when comparing polar group 3 with its closely related genomes in the same clade. This is further supported by the fact that the distribution of genome-wide GC content also showed the same profile, although whether the decrease in genome GC content is a good proxy for cold adaption remains to be validated.

The cold adaptation revealed by the genomic proxy agrees well with the growth temperature profiles, as polar 2 strains had lower minimum, optimum and maximum growth temperatures than the non-polar genomes. With the exception of the Arctic isolate, the decrease in growth temperature was also observed in polar group 3, but the phenotype did not match the genotype. Both genetic change and phenotypic plasticity play important roles in the environmental adaptation of bacteria. From the evidence of genomic and phenotypic traits, it is suggested that polar group 2 represents the true cold-adapted ecological unit in *Deinococcus*, while phenotypic plasticity facilitates the survival of polar group 3 in cold environments. Indeed, the trend of a downward shift in the growth temperature range was observed for most of the glacial bacteria [9, 14]. A recent study found that temperature adaptation had a strong phylogenetic signal and was vertically inherited in *Cryobacterium*, suggesting that the cold adapted lineages would form monophyletic clade [32].

Based on the observed phenotypic and genomic clues and ecological principle, we propose that the polar group 2 (*D. psychrotolerans* S14-83, *D. detaillensis* H1, *D. alpinitundrae* LMG 24283) is the genetically cold adapted clade in *Deinococcus*. The origin of polar group 2 may have undergone phenotypic adaptation followed by genetic adaptation, and it is also possible that it evolved directly from a mesophilic ancestor via genetic selection; but the true evolutional trajectory of the cold adapted lineage remains to be verified. For polar group 3, the phenotypic plasticity of downshifting growth temperature was not a stepping stone to its genetic adaptation to low temperature conditions. Vertically inherited genetic material and environmental conditions jointly influence microbial phenotype, and our study presents a case for distinguishing between the two [33, 34].

### Ecology of the cold adapted Deinococcus clade

Universal pathways or cellular processes for cold adaptation have not yet been identified. However, the definition of cold adapted clade (only refer to the polar group 2 hereafter) would enable the determining the genomic traits of *Deinococcus*-specific pathways or functions. The cold adapted clade had a similar functional distribution which was constrained by its taxonomy [35]. However, this cold adapted clade was significantly enriched in genes that involved in regulation and cell signalling, suggesting that the regulation of cellular processes is also important while the low temperature protein activity been achieved via amino acid optimization [11, 21, 36].

We then constructed the genetic potentiality of carbon, nitrogen, sulphur and arsenate cycle driven by *Deinococcus* to investigate the role of the cold adapted clade in biogeochemical cycling. The carbon cycle scheme indicated that the cold adapted clade are typical heterotrophic microorganisms that use organic carbon, including acetate and ethanol, as their main energy source, and are not able to fix nitrogen. The cold adapted *Deinococcus* would mainly act as CO_2_ producers in the polar and alpine environments. In the nitrogen cycle, the cold adapted clade is characterised as all of the four strains being able to conduct nitrite ammonification, while this function was shared by about 60% of other *Deinococcus*, suggesting that the cold adapted *Deinococcus* were enhanced in increasing the bioavailability of nitrogen, a common good of the community [37]. In the sulphur cycle, *Deinococcus* were predicted to be able to conduct the transformation between SO_3_^2−^ and SO_4_^2−^, and small proportion (10%, not including the cold adapted strains) were likely to be able to oxidise S^0^ to SO_3_^2−^. In the arsenate cycle, *Deinococcus* were predicted to be more preferred to reduce As^5+^ to As^3+^, suggesting that the mesophilic and psychrophilic *Deinococcus* are the protentional contributor of acutely toxic arsenate in temperate and cold environments [38]. In addition to the clade-specific functions, the other main ecological signature of cold adapted *Deinococcus* is that they could perform the main function of this genus at low temperatures; otherwise, the function would be lost according to the black queen and genome streamlining theories [37, 39, 40].

## Conclusion

In this study, we ordered the eight stains isolated from polar and alpine environment into three groups based on their genetic and phenotypic response to low temperature conditions: the polar group 1, refereeing to strain *D. arcticus* OD32, which processed no genetic chance and no phenotypic change; the polar group 2, refereeing to strains *D. marmoris* PAMC 26562, *D. marmoris* DSM 12784, *Deinococcus* sp. AJ005AJ005, *D. frigens* DSM 12807, which processed genetic change and downshifting growth temperature; the polar group 3, refereeing to strains *D. psychrotolerans* S14-83, *D. detaillensis* H1, *D. alpinitundrae* LMG 24283, which processed no genetic chance but downshifting growth temperature. Hence, we propose that polar group 2 represents the cold adapted ecotype of *Deinococcus*. Being able to cluster multiple isolates into cohesive ecological units facilitates the identification of genomic traits that are statistically associated with given environmental conditions [1, 21], which in turn will allow for a better understanding of how microbial communities respond to different environmental conditions in a changing world.

## Methods

### Strain isolation and genome sequencing

Strain *D. psychrotolerans* S14-83 was isolated from soil on the South Shetland Islands, Antarctica (62° 22′ 34″ S, 59° 42′ 34″ W) [17]. Strain *D. rufus* W37 was recovered from the type material deposited by Wang et al. [41] in CCTCC (China Center for Type Culture Collection). Genomic DNA was extracted from isolates using a TIANamp Bacteria DNA Kit (Tiangen, Beijing) following the manufacturer’s instructions. Using genomic DNA (extracted as described above) for the two isolates, paired-end libraries with an insert size of 500 bp were constructed and sequenced using an Illumina Hiseq 2000 platform. Filtered sequencing reads were subjected to assembly using SPAdes v3.11.1 with default options. The assembled genome sequences have been deposited in DDBJ/ENA/GenBank under the accessions PRJNA862670 [42] and PRJNA505982 [43].

### Preparation of Deinococcus genomes for analysis

In November 2021, all genome sequences with the taxonomic identifier ‘Deinococcus’ were retrieved from GenBank, providing a total of 134 genomes. As the taxonomy of this group is not well resolved, the taxonomy of the raw genomes was reclassified using GTDB-Tk [44]. The completeness and contamination of each taxonomically conformed genome were calculated using CheckM v1.0.7 with default options [22]. Genomes composed of > 300 contigs, with an N50 of < 20 kb, completeness of < 95% and contamination of > 5%, were removed. Genomes were dereplicated to remove genomes with an average amino acid identity (AAI) of ≥ 99.5%. AAI values were calculated by CompareM with default options (https://github.com/dparks1134/CompareM). Ultimately, there were 85 genomes that met the quality requirements, including the two genomes sequenced in this study. Detailed information concerning the origin, biogeography and genomic quality of the data is provided in Table S1.

### Phylogenetic and genomic analyses

Outgroup species that are closely related to the ingroup species are more suitable for phylogenetic reconstruction than distantly related species or ingroup species [45]. Thus, for phylogenomic clustering, complete genomes of *Meiothermus cerbereus* DSM 11376 (GCA_000620065.1) and *Meiothermus* sp. QL-1 (GCA_003351145.1) were chosen as the outgroup species as they are close relatives of *Deinococcus* [46]. With the two *Meiothermus* and 85 *Deinococcus* genomes, a maximum likelihood phylogenomic tree was constructed using PhyloPhlAn3 with default options [47]. As the phylogenomic tree can be drawn in multiple different equivalent appearances, to obtain a relatively fixed phylogenetic topology, the tree was sorted with increasing node order using FigTree 1.4.4 (https://github.com/rambaut/figtree/releases).

The annotation of genes was standardised by annotating all genomes using PROKKA v1.14.5 with default options [48]. Genomic-scale reconstructions of metabolic pathways and biogeochemistry profiles were performed with gapseq v1.2 [49] and METABOLIC v4.0 [24]. The pan-genome of *Deinococcus* was constructed using PEPPAN v1.0.5 with default options and the gff files produced by PROKKA as the input. The result produced by the main program of PEPPAN was parsed using PEPPAN_parser with the arguments -t -c -a 95 and leaving others as their defaults [50]. Rarefaction curves of the pan and core genes were visualised with a custom made R script [51]. ANI was calculated using the ANI calculator (http://enve-omics.ce.gatech.edu/ani/). AAI values were calculated using CompareM with default options (https://github.com/dparks1134/CompareM).

### Supplementary Information

Additional file 1: Table S1. Information of *Deinococcus* genomes used in this study.

Additional file 2: Figures S1-S2. Fig. S1. Cladogram and dot plots showing the genome-wide GC content, genome size, and predicted complete metabolic pathways in *Deinococcus*. Fig. S2. Comparison of GC contents between *Deinococcus* groups.

## Data Availability

The assembled genome sequences have been deposited in DDBJ/ENA/GenBank under the accessions PRJNA862670 [42] and PRJNA505982 [43].

## Abbreviations

MAGs: Metagenome-assembled genomes
AAI: Average amino acid identity
ANI: Average nucleotide identity
