# Supplementary material for: Genomics-based identification of a cold adapted clade in Deinococcus
Source: BMC Biol. 2024 Jul 2;22:145. doi: 10.1186/s12915-024-01944-8 (PMC11218099; doi:10.1186/s12915-024-01944-8)

Genomics-based identification of a cold adapted clade in *Deinococcus*

Liang Shen^1, 2*^, Jiayu Hu^1^, Luyao Zhang^1^, Zirui Wu^1^, Liangzhong Chen^1^, Namita Paudel Adhikari^3,4^, Mukan Ji^4^, Shaoxing Chen^1^, Fang Peng^5*^, Yongqin Liu^3,4^

^1^College of Life Sciences, Anhui Normal University, Wuhu 241000, China

^2^Anhui Provincial Key Laboratory of Molecular Enzymology and Mechanism of Major Diseases, and Auhui Provincial Engineering Research Centre for Molecular Detection and Diagnostics, Anhui Normal University, Wuhu 241000, China

^3^State Key Laboratory of Tibetan Plateau Earth System, Environment and Resources (TPESER), Institute of Tibetan Plateau Research, Chinese Academy of Sciences, Beijing 100101, China

^4^Center for the Pan-third Pole Environment, Lanzhou University, Lanzhou 730000, China

^5^China Center for Type Culture Collection (CCTCC), College of Life Sciences, Wuhan University, Wuhan 430072, PR China

*Correspondence: Liang Shen, shenliang@ahnu.edu.cn; Fang Peng, pf-cctcc@whu.edu.cn.

Supplementary Figures

Fig. S1 **Cladogram and dot plots showing the genome-wide GC content, genome size, and predicted complete metabolic pathways in *Deinococcus***. Isolates in the root part of the tree had the lowest GC content (~56%), while those at the top had the highest content (~71%); i.e., the variation in genome GC content shows a trend of convergence from the root to the top of the tree. The genome size of *Deinococcus* ranged from 2.46 Mb to 6.65 Mb, and the isolates with the smallest and largest genomes were all located in the lower part of the tree. The predicted complete metabolic pathways ranged from 206 to 300, with isolates conducting more metabolic reactions concentrated in the upper part of the tree.

Fig. S2 **Comparison of GC contents between *Deinococcus* groups**. ^‡^This non-polar group including six genomes, *Deinococcus* sp. D7000, *D. radiopugnans* DY59, *D. radiopugnans* ATCC 19172, *D. humi* DSM 27939, *D. aerolatus* JCM 15442 and *D. aerophilus* JCM 15443; ^†^this non-polar group including four genomes, *D. irradiatisoli* 17bor-2, *Deinococcus* sp. bin10, and *D. aquiradiocola* JCM 14371.

Fig. S1

Fig. S2


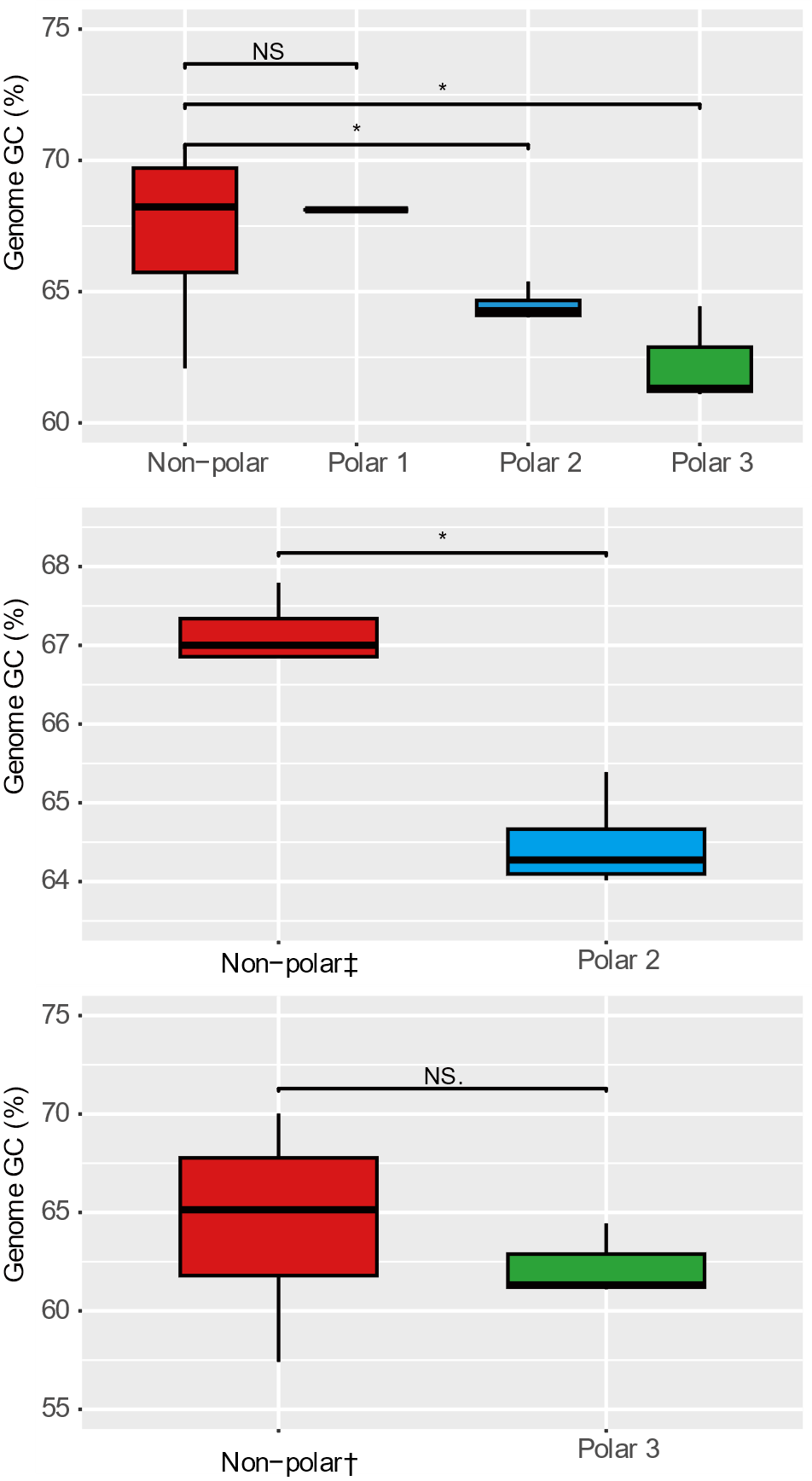

Supplement: Supplementary file 2 — Additional file 2: Figures S1-S2. Fig. S1. Cladogram and dot plots showing the genome-wide GC content, genome size, and predicted complete metabolic pathways in Deinococcus. Fig. S2. Comparison of GC contents between Deinococcus groups. [file 12915_2024_1944_MOESM2_ESM.docx]
